# Deregulation of IGF-binding proteins -2 and -5 contributes to the development of endocrine resistant breast cancer *in vitro*

**DOI:** 10.18632/oncotarget.8534

**Published:** 2016-04-01

**Authors:** Yousef Hawsawi, Matthew P. Humphries, Alexander Wright, Angelene Berwick, Mike Shires, Hanaa Al-Kharobi, Reem El-Gendy, Maria Jove, Chris Twelves, Valerie Speirs, James Beattie

**Affiliations:** ^1^ Department of Oral Biology, St James's University Hospital, Leeds, UK; ^2^ St James's Institute of Oncology, St James's University Hospital, Leeds, UK; ^3^ Leeds Institute of Cancer and Pathology, University of Leeds, UK; ^4^ Current address: Department of Breast Medical Oncology, MD Anderson Cancer Centre, University of Texas, Houston, USA

**Keywords:** IGF, breast cancer, endocrine resistance

## Abstract

Tamoxifen (TAM) remains the adjuvant therapy of choice for pre-menopausal women with ERα-positive breast cancer. Resistance and recurrence remain, however, a major challenge with many women relapsing and subsequently dying. The insulin-like growth factor (IGF) axis is involved in breast cancer pathogenesis and progression to endocrine resistant disease, but there is very little data on the expression and potential role of IGF-binding proteins (IGFBP) during acquisition of the resistant phenotype. The aim of this study was to determine the expression and functional role of IGFBP-2 and -5 in the development of TAM resistance (TamR) *in vitro* and to test retrospectively whether they were predictive of resistance in a tissue microarray of 77 women with primary breast cancers who relapsed on/after endocrine therapy and 193 who did not with long term follow up. Reciprocal expression of IGFBP-2 and IGFBP-5 was observed at both mRNA and protein level in TamR cells. IGFBP-2 expression was increased by 10-fold while IGFBP-5 was decreased by 100-fold, compared to TAM-sensitive control cells. shRNA-mediated silencing of IGFBP-2 in TamR cells restored TAM sensitivity suggesting a causal role for this gene in TamR. While silencing of IGFBP-5 in control cells had no effect on TAM sensitivity, it significantly increased the migratory capacity of these cells. Quantitative image analysis of immunohistochemical data failed, however, to demonstrate an effect of IGFBP2 expression in endocrine-relapsed patients. Likewise, IGFBP-2 and IGFBP-5 expression failed to show any significant associations with survival either in patients relapsing or those not relapsing on/after endocrine therapy. By contrast, *in silico* mining of a separate published dataset showed that in patients who received endocrine treatment, loss of expression of IGBP-5 was significantly associated with worse survival. Overall these data suggest that co-ordinated and reciprocal alteration in IGFBP-2 and −5 expression may play a role in the acquisition of endocrine resistance.

## INTRODUCTION

Development of resistance to anti-oestrogen therapies in patients with ER+ breast cancer (BC) represents a major therapeutic challenge. This is particularly evident in the eventual failure of adjuvant tamoxifen in a proportion of patients, particularly in the premenopausal setting where it remains the therapy of choice. Although there is growing evidence that other signalling pathways provide an alternative mitogenic drive to epithelial tumour cells resistant to tamoxifen [1], the precise molecular detail of such mechanisms remains unclear. The insulin-like growth factor (IGF) axis plays an important role in the development of normal mammary gland where, in concert with 17β oestradiol, it regulates the proliferation, differentiation and apoptosis of mammary epithelial cells during pregnancy, lactation and subsequent involution of the gland [2]. It has been suggested that deregulation of the IGF axis may be involved in BC initiation, development and subsequent metastasis especially with respect to underlying tamoxifen resistance. Some studies have reported an increased expression of IGF-1R following acquisition of TamR [3–6] with the inference that such an increase in cell membrane receptor density may lead to an increased sensitivity to IGF ligands. In many instances, however, such causal relationships have not been validated and subsequent studies have reported no alterations or even down regulation of IGF-1R expression in TamR cells [7–9].

These findings are consistent with the general failure of anti-IGF-1R based strategies in clinical trials and have led to an interest in the expression and activity of other IGF axis components as possible targets in developing anti-tumour strategies. Principal amongst these have been an examination of IGFBP expression and activity in primary BC cells and cell lines. Expression of IGFBPs by breast tissue and cell lines is extensively documented [10, 11] and they are believed to regulate access of stromal derived IGF-1 and -2 to epithelial cell IGF-1R. However IGFBPs are also reported to have an IGF enhancing effect in breast cancer tissues and also to have direct effects that are independent of IGFs [12–14]. These pleiotropic actions of IGFBP in breast tissue, and the association of the IGF axis with an alternative mitogenic stimulus in TamR breast cancer cells, identify IGFBPs as potential alternative targets for anti-IGF axis therapeutic strategies.

Because there is very little data on the expression and role of IGFBPs during the acquisition of TamR we have examined (i) the expression and activity of IGFBPs in the ER+ MCF-7 cell line, (ii) the role of these genes in the development of TamR and the migratory phenotype and (iii) retrospectively tested whether IGFBPs expression and activity was predictive of resistance in a cohort of patients with breast cancer and with long term follow up.

## RESULTS

**Figure 1:**
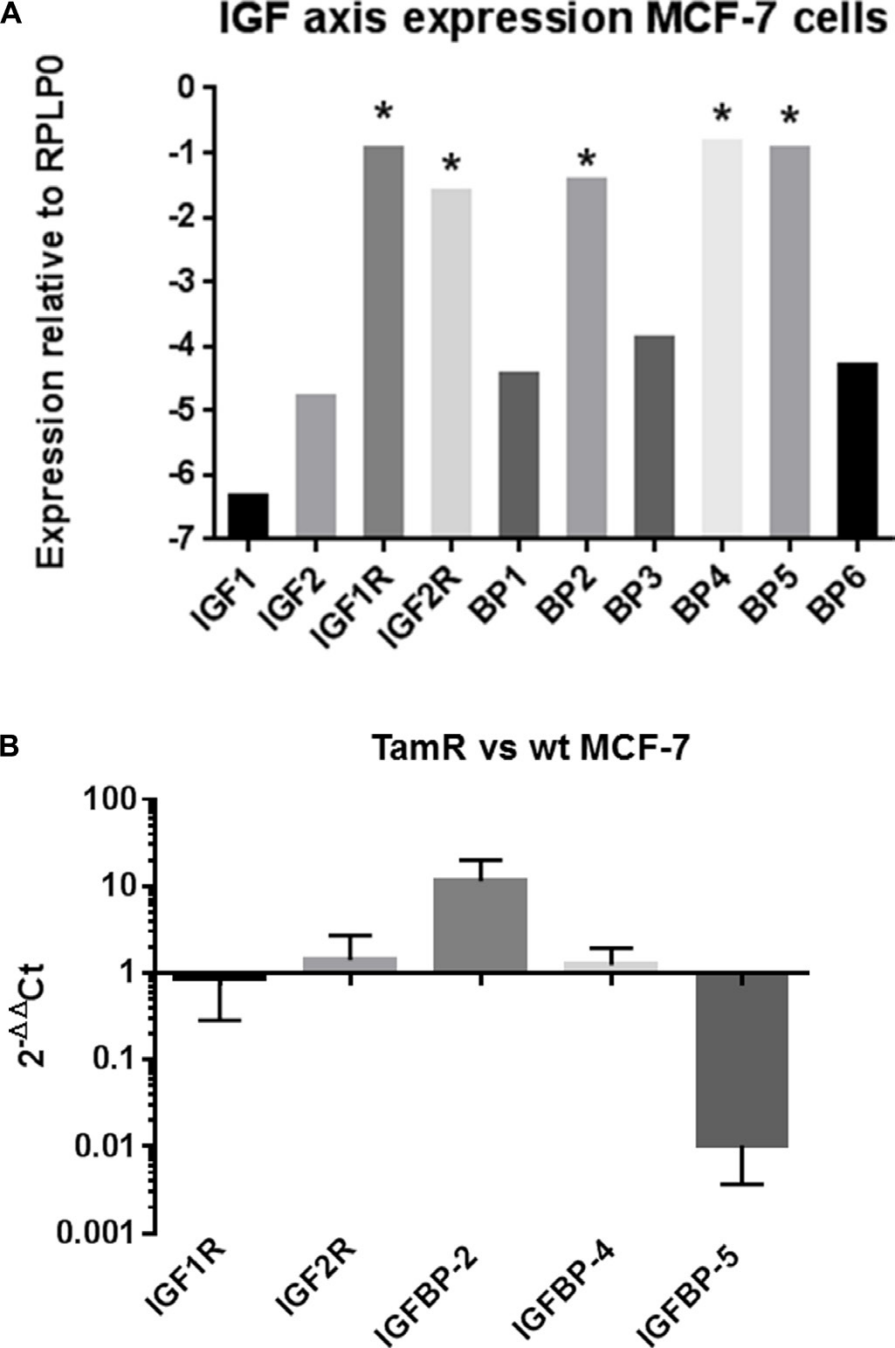
(**A**) Expression of 10 IGF axis genes in MCF-7 cells. Expression is plotted on a logarithmic scale relative to the house keeping gene RPLP0 and is represented as 2^−ΔCt^ (see Materials and Methods section for further details). Moderate to highly expressed genes are indicated (*). (**B**) Expression of IGF1R, IGF2R, IGFBP-2, −4 and −5 in wt and TamR MCF-7 cells. Data is plotted as 2^−ΔΔCt^ and represents the fold change in gene expression TamR v wt MCF-7 cells. This experiment was repeated four times with duplicate technical repeats. Data is presented as mean ± SD (*n* = 4).

**Figure 2:**
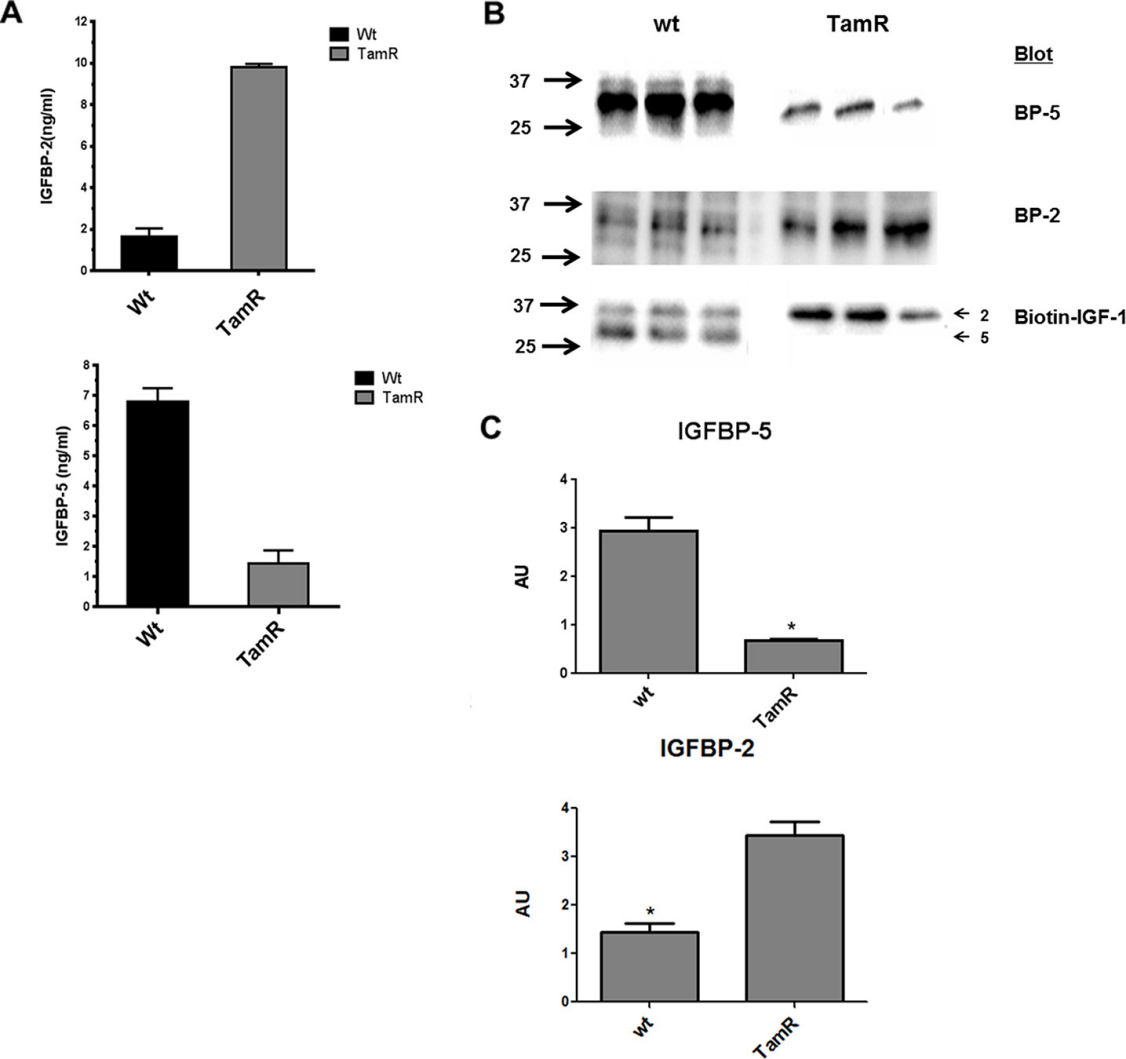
(**A**) Elisa determination of IGFBP-2 and -5 concentrations in conditioned medium (CM) from wt and TamR MCF-7 cell lines. Data represent mean ± SD (*n* = 3). Experiments were repeated on three separate occasions. *p < 0.001, **p < 0.0001 (Student's unpaired *t*-test). (**B**) Western blot (upper two panels) and Ligand blot (lower panel) identifies IGFBP-2 (upper arrow Ligand blot) and IGFBP-5 (lower panel Ligand blot) in CM from wt and TamR MCF-7 cells. (**C**) Densitometric analysis of Western blots for IGFBP-2 (upper graph) and IGFBP-5 (lower graph) band intensity. Triplicate lanes per blot were analysed for both wt and TamR conditioned media using Image Lab software. Data are presented as mean +/− SD (*n* = 3) of Arbitrary Unit (AU) intensity. This experiment was repeated 3 times with similar results in each instance. *p < 0.005 TamR v MCF-7 Students *t*-test. GraphPad Prism 5.0.

Five of the ten genes of the IGF axis interrogated, IGF1R, IGF2R, IGFBP-2, -4 and -5 were expressed at moderate to high levels by both wt and TamR MCF- 7 cells. The other five genes IGF-1, IGF-2, IGFBP-1, IGFBP-3 and IGFBP-6 were expressed at very low levels – note the logarithmic scale in Figure 1A. Repeated qRT-PCR analysis on different batches of cells indicated reciprocal regulation of IGFBP-2 and -5 in TamR versus wt cells such that IGFBP-2 expression was up regulated approximately 10-fold whereas IGFBP-5 expression was down regulated approximately 100-fold in TamR cells (Figure 1B). Expression of IGF1R, IGF2R or IGFBP-4 did not significantly differ between TamR and wt cells. Changes in IGFBP-2 and -5 mRNA concentrations were reflected in protein concentrations in wt and TamR cell conditioned medium (CM) as determined by ELISA (Figure 2A), Western blot (Figure 2B, upper two panels) or IGF ligand blot (Figure 2B, lower panel). Therefore ELISA indicated IGFBP-2 concentrations of 1.7 ± 0.69 ng/ml and 9.8 ± 0.24 ng/ml (mean ± SD *n* = 3) for wt and TamR CM respectively and IGFBP-5 concentrations of 6.8 ± 0.78 and 1.4 ± 0.75 ng/ml (mean ± SD, *n* = 3) both *p* < 0.0001 wt v TamR. These differences were confirmed by densitometric analysis of Western blots (Figure 2C). In some instances for ligand blot of TamR CM IGFBP-5 was below the detection level for this technique – Figure 2 bottom panel (lower arrow).

**Figure 3:**
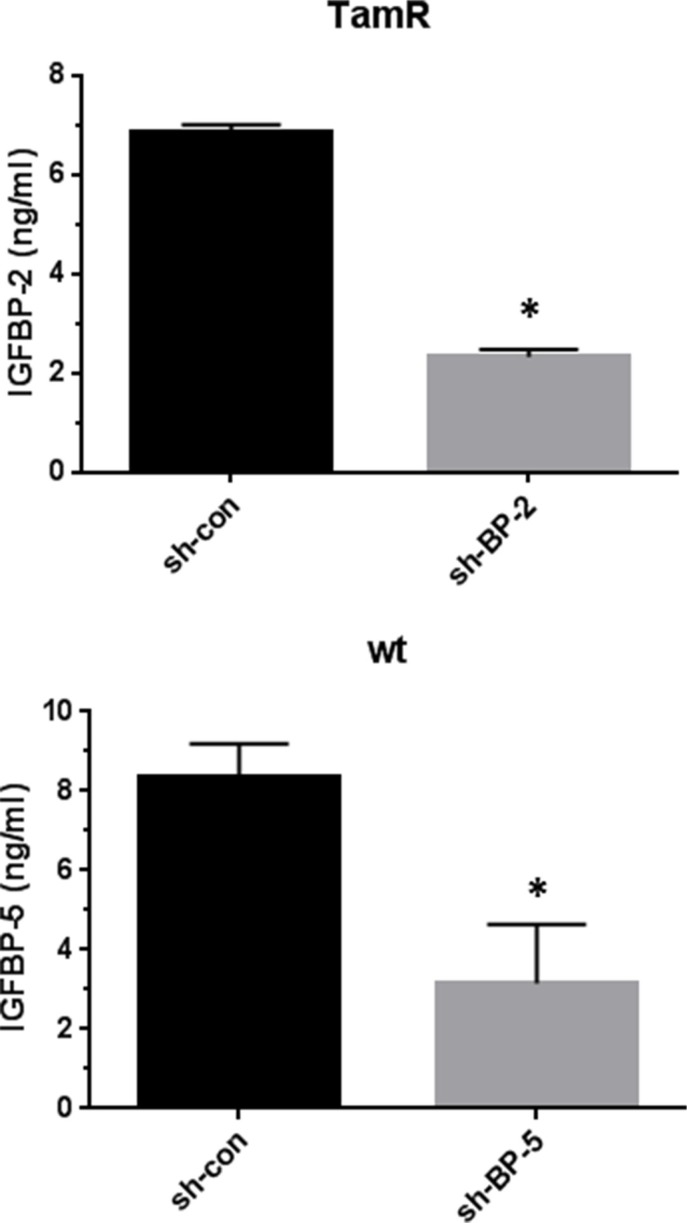
The knockdown of IGFBP-2 expression in TamR cells and IGFBP-5 expression in wt MCF-7 cells Conditioned medium from clone F8 (TamR/BP2 knockdown) and scrambled sequence control transfected TamR cells was assayed for IGFBP-2 by Elisa (upper panel). Conditioned medium from clone B4 (wtMCF-7/BP5 knockdown) and scrambled sequence control transfected cells was assayed for IGFBP-5 by Elisa (lower panel). Data are expressed as mean ± SD *n* = 3. Columns with different superscripts are statistically different *p < 0.05 Student's unpaired *t*-test; GraphPad Prism 5.0.

**Figure 4:**
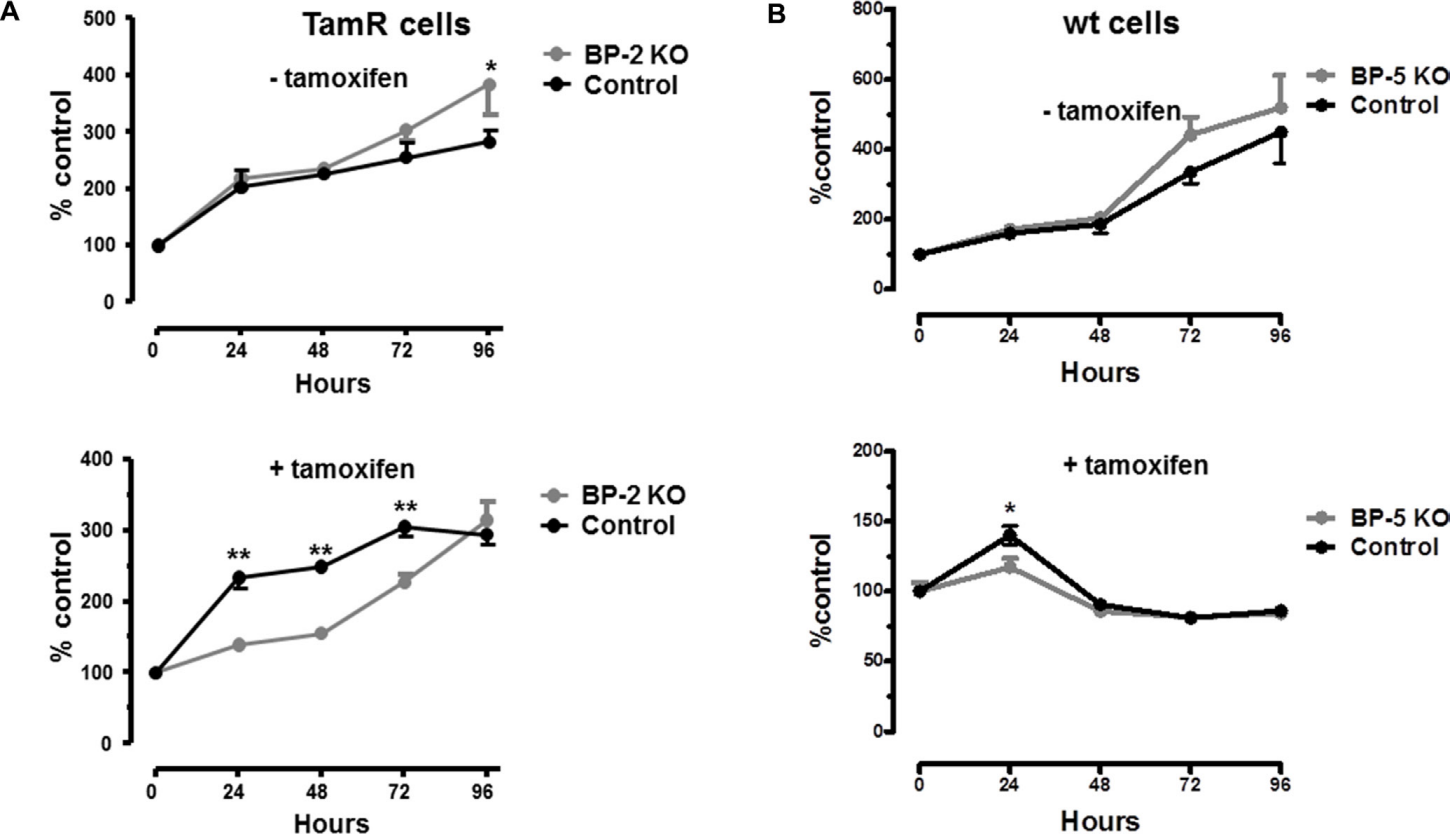
(**A**) Growth of TamR BP-2 KO clone F8 or scrambled control cells in the absence (top panel) or presence (bottom panel) of 1 uM 4 HT. Cell growth was monitored over the period 0–96 hr. by WST-1 assay as described in Materials and Methods and was normalised to *t* = 0 (100%). This experiment was repeated five times with three technical repeats in each experiment and data is presented as mean ± SD (*n* = 5). In some instances SDs are smaller than the size of the symbol. Curves were analysed by repeated measures ANOVA followed by Bonferroni's post-hoc test *p < 0.05 **p < 0.001 GraphPad Prism 5.0. (**B**) Growth of WtMCF-7 clone (B4) or scrambled control cells in the absence (top panel) or presence (bottom panel) of 1 uM 4 HT. Cell growth was monitored over the period 0–96 hr. by WST-1 assay as described in Materials and Methods and was normalised to *t* = 0 (100%). This experiment was repeated five times with three technical repeats in each experiment and data is presented as mean ± SD (*n* = 5). In some instances SDs are smaller than the size of the symbol. Curves were analysed by repeated measures ANOVA followed by Bonferroni's post-hoc test *p < 0.05 **p < 0.001 GraphPad Prism 5.0.

To examine whether differences in IGFBP-2 or -5 expression were associated with the development of TamR in MCF-7 cells IGFBP-2 was shRNA silenced in TamR cells to examine whether this resulted in increased sensitivity to 4 HT. Similarly, IGFBP-5 was silenced in wt MCF-7 cells to examine whether this induced tamoxifen resistance. Scrambled shRNA served as an experimental control. Typically, 8–12 clones showed successful knock down for IGFBP-2 and IGFBP-5. Figure 3 shows IGFBP-2 and IGFBP-5 levels in conditioned medium for two stably transfected clones F8 (IGFBP-2) and B4 (IGFBP-5). Knockdown of over 60% as determined by ELISA was achieved in both instances. This established these clonal cell lines as appropriate models for examination of a causative role for IGFBP-2 and IGFBP-5 in the development of tamoxifen resistance in MCF-7 cells. When BP2 silenced and control transfected TamR cells were grown in the absence of 4 HT there was little difference in the growth curves for the cells with only the 96 hr time point showing a statistically significant difference (*p* < 0.05 BP-2 silenced v control-Figure 4A, top panel). However, when cells were incubated in the presence of 1 μM 4 HT then growth of BP2 silenced cells was significantly compromised compared to that of control cells at 24, 48 and 72 hr time points (*p* < 0.001). This suggests that knock down of IGFBP-2 expression to levels approaching those seen for wt MCF-7 cells partially restores sensitivity of cells to 4 HT. For IGFBP-5 knock down in wt MCF-7 cells no difference in the growth curves for silenced BP5 v control cells was seen in the absence of 4 HT (Figure 4B-top panel). However in the presence of 1 μM 4 HT the growth of both cell lines was inhibited (Figure 4B, bottom panel). This suggests that the knock down of IGFBP-5 to levels seen in TamR cells does not confer tamoxifen resistance to wt MCF-7 cells.

**Figure 5:**
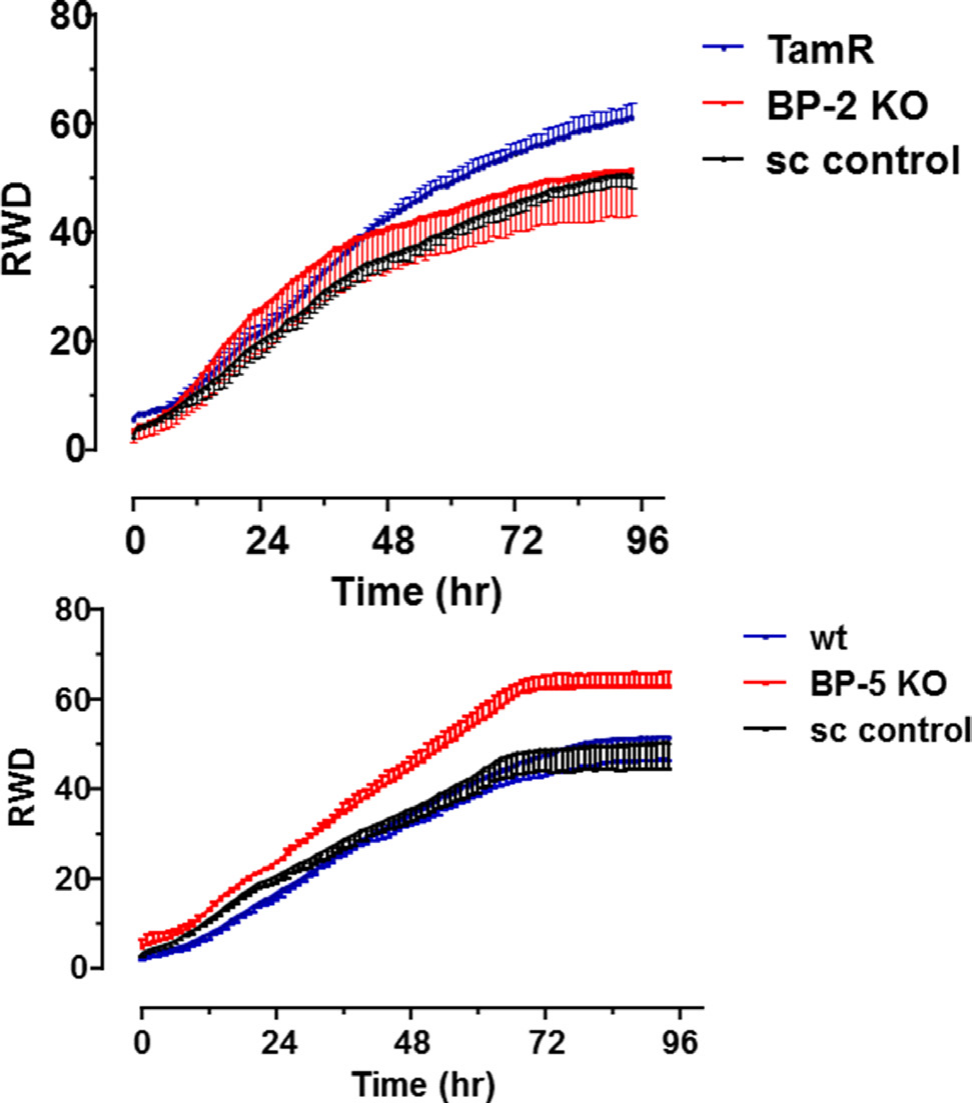
Migration of IGFBP knockdown cells plotted as relative wound density (RWD) v time Top panel – untransfected TamR cells (TamR), clone F8 (BP-2 KO) or scrambled shRNA (sc control) transfected cells were seeded and wound closure was measured in real time by Incucyte as described in Materials and Methods. Bottom panel- untransfected wt MCF-7 cells (wt), clone B4 (BP-5 KO) or scrambled shRNA (sc control). Cell migration was monitored over the period 0–96 hr. Data are presented as mean ± SD; *n* = 12 for each time point. This experiment was performed twice with similar results in each instance and a representative experiment is shown. Curves were analysed by two way ANOVA followed by Bonferroni's post-hoc test. *p < 0.0001 BP-5 KO v wt or sc control.

The development of TamR in breast cancer epithelial cells is often associated with increased cell migration. Using IncuCyte real time wound healing methodology we confirmed that TamR cells showed more rapid migration than wt MCF-7 cells (Supplementary Figure S1). Against this background we examined whether sh-BP cell lines had altered migration properties with respect to parental wt or TamR cells. For BP2 silenced cells migration was significantly lower than that for parental TamR cells suggesting that IGFBP-2 knock down inhibited cell migration (Figure 5, top panel). However there was no significant difference between sh-BP2 and sh-control transfected cells with respect to cell migration profile suggesting that any effects which were seen were non-specific. However for BP5 silenced cells there was a specific increase in cell migration evident when using Incucyte methodology (Figure 5, bottom panel) suggesting that knock down of IGFBP-5 in wt MCF-7 cells increased cell migration, resembling the TamR phenotype (Supplementary Figure S1) and that IGFBP-5 therefore may act as an inhibitor of cell migration.

**Figure 6:**
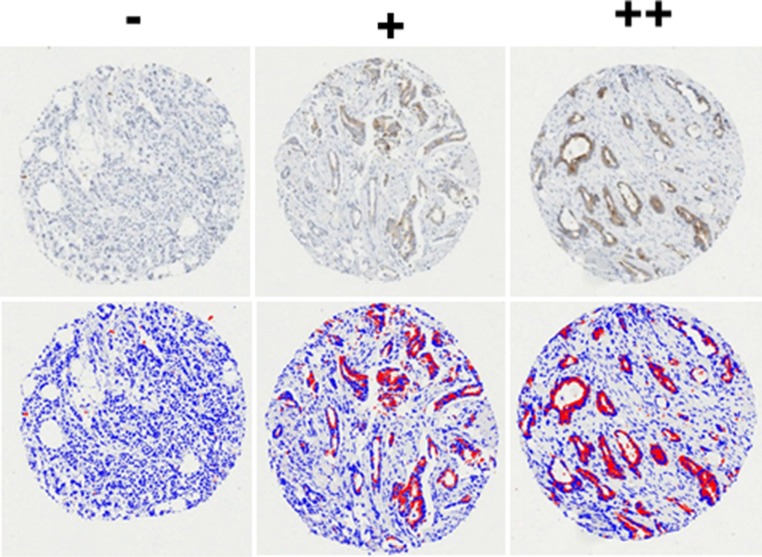
Representative tissue cores showing cytoplasmic staining that represents expression of IGFBP-2 in breast cancer The slides were scanned at x20 magnification using the ScanScope™ system, and visualised for scoring using the ImageScope™ pixel intensity Aperio algorithm. Before application of the algorithm, the blue indicates the negative and the brown represents the positive. After application of the algorithm, the blue indicates the negative and the red represents the positive. Examples of negative (−), moderately positive (+) and strongly positive (++) cores are shown.

**Figure 7:**
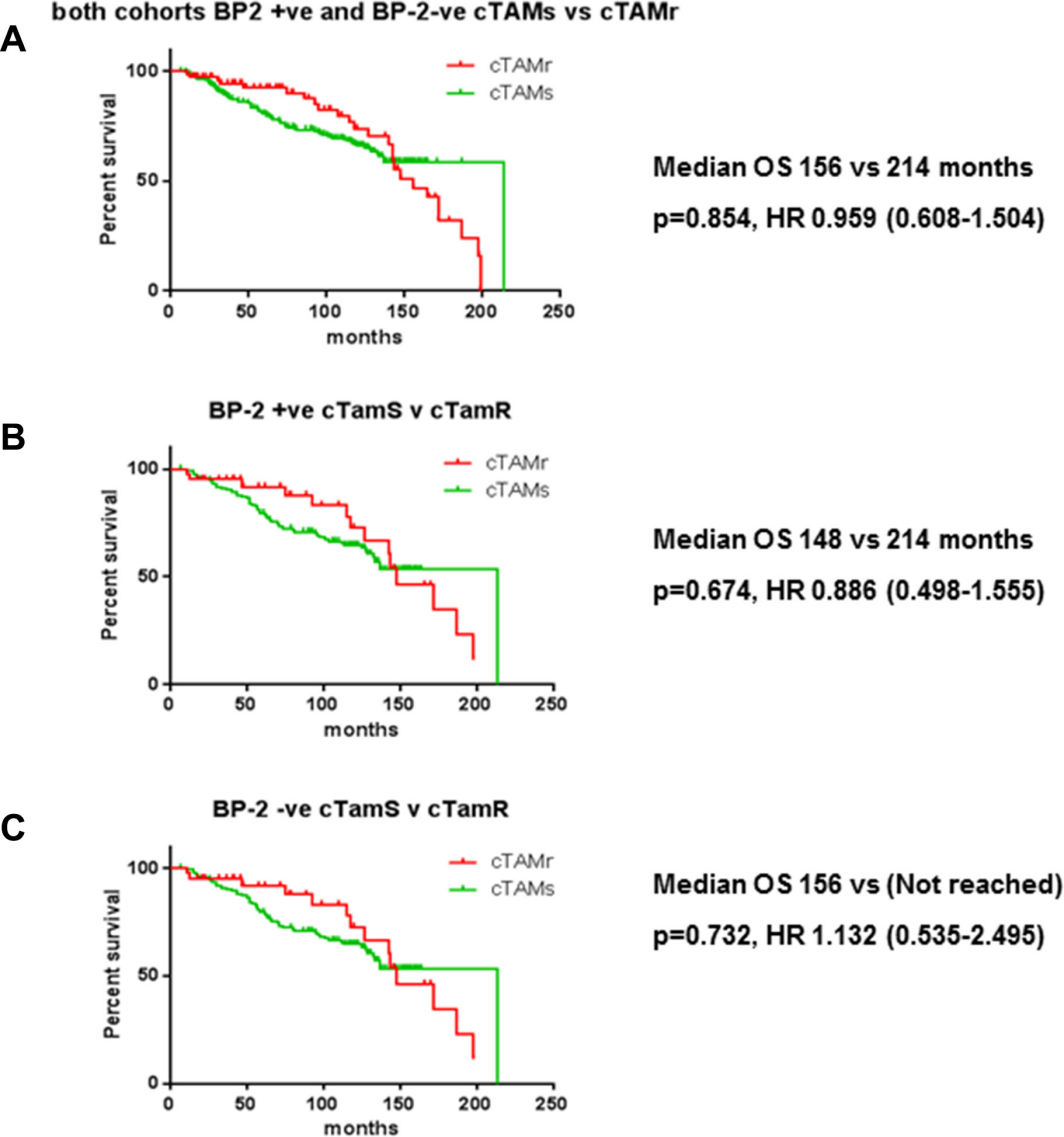

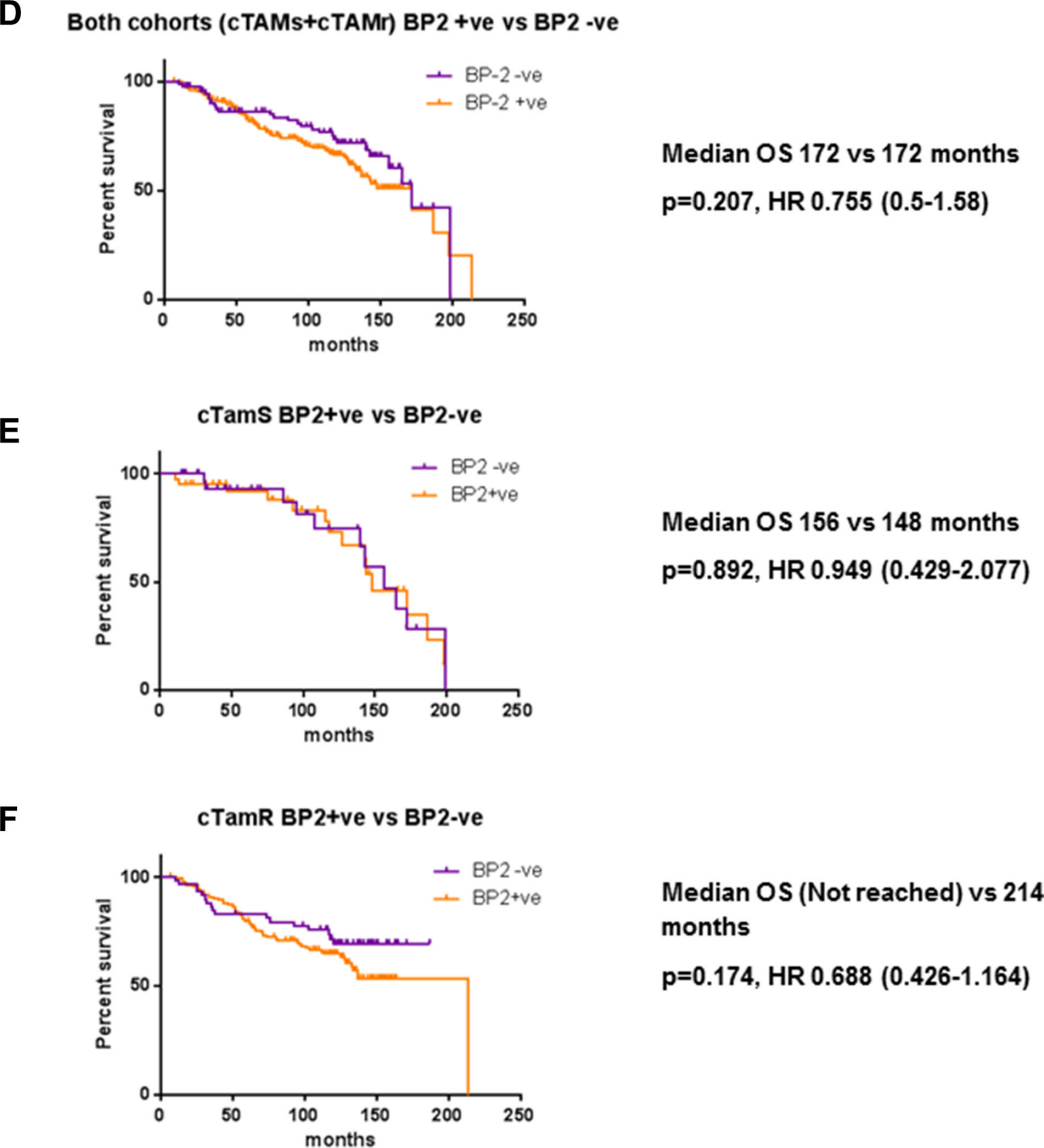
Kaplan-Meier survival curves for both clinically tamoxifen resistant (cTamR) and clinically tamoxifen sensitive (cTamS) patients stratified according to IGFBP2 status (A–C) or tamoxifen sensitivity (D–F) Whole cohort stratified for IGFBP2 status (a), cTamR (b), cTamS (c), whole cohort stratified for tamoxifen sensitivity (d) cTamR (e), cTamS (f). Data were analysed with Univariate Cox regression and *p* values and Hazard ratios are shown next to relevant panels.

Because of the association of IGFBP-2 with tamoxifen resistance, we next asked whether IGFBP-2 expression might be a useful marker of tamoxifen resistance using a cohort of clinical samples - the relationship between IGFBP-2 expression and clinico-pathological parameters is shown in Supplementary Table S1(A + B). We used image analysis to quantify expression of both of IGFBP-2 staining before and after application of the algorithm is shown in Figure 6 and described in the accompanying figure legend. Kaplan-Meier analysis indicated no significant survival differences between IGFBP-2 positive and IGFBP-2 negative tissues in either the non-stratified cohort or those stratified for endocrine sensitivity (cTamR and cTamS; Figure 7A–7F). The same analysis was applied to cases stained for IGFBP-5; again no statistically significant findings were apparent (data not shown).

*In silico* analysis of data mined from Kaplan Meier Plotter in patients who received endocrine treatment showed that loss of expression of IGBP-5 at the mRNA level was associated with significantly worse overall survival (*p* = 0.0075; HR = 0.3, range 0.11–0.76, Supplementary Figure S2). No such relationship was seen with respect to IGBP-2 expression HR 1.03, range 0.44–2.42. (data not shown).

## DISCUSSION

This work set out to examine the contribution of the IGF axis, specifically IGFBP-2 and -5 to the development of endocrine resistant breast cancer. We found 5 of the 10 IGF axis genes were expressed by MCF-7 cells at moderate or high levels confirming previous reports for this cell line and other ER+ BC cell lines [15, 16]. Although 5 of the IGF axis genes are expressed only at low levels this does not infer that they play an insignificant role in BC [17]. The up regulation of IGFBP-2 following the development of TamR in MCF-7 cells has been reported previously [18] although siRNA experiments by these authors suggested that IGFBP-2 was a marker for development of TamR but was not a causative factor in this process. This is in contrast to our current observations where stably transfected IGFBP-2 knock down cells clearly exhibited increased sensitivity to 4 HT. These differences may be explained by transient transfection as opposed to stable transfection protocols used in the current study. It is important to note that at later time points (96 hr) sensitivity to 4 HT is overcome (Figure 4A – lower panel). One explanation for this is that IGFBP-2 may accumulate both intra- and extracellularly with time and reach a threshold value in culture which restores 4 HT resistance. However this possibility requires rigorous investigation.

IGFBP-5 knock down in wt MCF-7 cells did not result in the development of TamR in these cells. Ahn et al. reported IGFBP-5 expression decreased in TamR MCF-7 cells but also used an RNA screening methodology to indicate that IGFBP-5 played a causal role in the determination of tamoxifen sensitivity [19]. They also demonstrated that shRNA based knockdown of IGFBP-5 expression in MCF- 7 cells conferred tamoxifen resistance in these cells and addition of exogenous IGFBP-5 partly restored sensitivity to tamoxifen. Although it is difficult to rationalise our findings with those described by Ahn et al. we note that shRNA knock down of IGFBP-5 expression led to a loss of ERα expression in the studies described by Ahn et al. Similarly Foulstone et al. using Western blotting techniques recently demonstrated that silencing IGFBP-2 expression ablated ERα expression in MCF-7 cells. Although this was not the case in our studies where ERα expression was not reduced in TamR BP2 KO cells compared to sc transfected cells or untransfected cells by qRT-PCR analysis (data not shown) this is an area of increasing interest and is worthy of further study.

The development of TamR is often associated with enhanced migration of resistant cells [20]. Although we found no specific effect of IGFBP-2 knock down on migration of TamR cells there is a literature which supports a role for IGFBP-2 in the regulation of MCF- 7 cell migration and associated phenotypes although it is in part conflicted. Therefore, IGFBP-2 has been shown to down-regulate PTEN expression by an integrin-mediated mechanism in MCF-7 cells culminating in a marked increase in cell proliferation [21]. However in MCF-7 cells over expressing integrin β3, IGFBP-2 inhibits IGF mediated cell migration an effect associated with integrin mediated localisation of IGFBP-2 to the cell surface [22]. An engineered protease resistant IGFBP-2 inhibits MCF-7 tumour cell growth as a xenograft in a female nude Balb/c mouse model illustrating the importance of post-translational modification on the activity of IGFBPs [23]. Pleiotropic effects of IGFBP-2 are also evident and in the ER -ve Hs578T cell line. IGFBP-2 promoted de- adhesion of cells but inhibited proliferation through an α5β1 integrin binding mechanism [24]. As this cell line lacks a functional IGF1R such effects were postulated to occur in an IGF-1 independent fashion and indeed subsequent studies using microarray analysis in Hs578T cells demonstrated that exogenous IGFBP-2 regulated the expression of several genes associated with cell proliferation, adhesion and apoptosis [25]. Our data were confounded by non-specific inhibition of cell migration in sh-con transfected cells arguing for non-specific off target effects in respect of this particular phenotype. Alternative sh-BP2 targeting vectors and/or sequences may help resolve some of the issues highlighted above.

In contrast to the results for IGFBP-2 knock down we found clear evidence that knock down of IGFBP-5 in wt MCF-7 cells was associated with increased cell migration and therefore resembled the phenotype of TamR cells where IGFBP-5 expression is reduced. These data are consistent with previous findings from our group which report that IGFBP-5 inhibits MCF-7 cell migration [26] and therefore any decrease in IGFBP-5 expression may be associated with increased migratory potential. IGFBP-5 also enhances adhesion of MCF-7 cells to mesenchymal cell derived matrix and may play a role in the inhibition of epithelial-mesenchymal transition (EMT) a process closely associated with tumour cell development and metastasis [27]. In contrast to our data, Kricker et al. reported that IGFBP-5 enhanced IGF- 1 stimulation of MCF-7 cell migration when cultures were grown on vitronectin [28] although a distinction must be made between IGF-dependant and independent effects of IGFBPs. We acknowledge our *in vitro* data may have limitations as this was generated from a single cell line, MCF-7 and its isogenic tamoxifen-resistant variant developed in house over 21 months [29, 30]. Nevertheless this is consistent with other work in this field where MCF- 7 is widely regarded as the most suitable cell line to study tamoxifen resistance due to its expression of ERα and exquisite hormone sensitivity *in vitro* [31], with recent publications using single MCF-7 derivatives [32, 33].

Our TMA analysis indicated that within both cTamS and cTamR cohorts of patients there was no significant association between IGFBP-2 (or IGFBP-5) expression and overall survival in the relatively modest numbers of samples available. Our *in silico* data mining analysis of a smaller, independent data set also failed to show a relationship between IGFBP-2 expression and OS. Evidence elsewhere for an association between IGFBP-2 expression and BC progression remains contradictory [34] with reports of association and non-association with overall survival or tumour progression [35–37]. Other studies have suggested that IGFBP-2 (and IGFBP-5) expression may provide prognostic or predictive value for BC [38], especially with respect to success of endocrine therapies [19, 39, 40]. An IHC study on resected BC tissues showed a gradual elevation in IGFBP-2 expression from atypical hyperplasia through to carcinoma *in situ* and invasive carcinoma [35] and a TMA analysis of more than 4,000 primary invasive breast tumours indicated that an adverse survival outcome is correlated with IGFBP-2 expression in ERα negative tumours [41]. IGFBP-2 expression combined with cell adhesion protein, β-catenin, is linked with lymph node metastasis in BCs [42] and high levels of IGFBP-2 expression together with loss of PTEN expression are also associated with triple negative (TN) BC and poor survival rates [43]. IGFBP-2 is up-regulated in an *in vitro* model of Her-2+ BC [44] and recently multiple antigenic peptides (MAPs) comprising IGFBP-2 epitopes have been used to block tumour progression in a transgenic mouse model [37, 45].

Although no clear relationship between high IGFBP-5 expression and overall survival was seen in our TMAs from cTamR and cTamS patients (with endocrine resistant and endocrine sensitive disease, respectively) we did observe a relationship when transcriptomic data were assessed using KMPlotter [23]. The reason for this apparent discordance is unclear, but there are important differences between the analyses. First, the IGFBP-5 analysis, defined as positive or negative, was conducted in patients defined as tamoxifen “sensitive” or “resistant”, whereas the *in silico* was analysis of all patients matching the selection criteria with IGFPB-5 expression defined as above or below the median. Secondly, there may be differences and/or inconsistences in the completeness or accuracy of the clinical data between the data sets. Finally, whereas in the TMA analysis IGFBP-5 data were available for a large proportion of the original population, the *in silico* analysis included only 65 of 3557 patients in the KMPlotter database.

Elsewhere, IGFBP-5 mRNA has been reported to be up-regulated in breast tumour tissue relative to normal mammary gland although there was no association between IGFBP-5 expression and tumour grade [46]. Reports suggest that IGFBP-5 is either elevated [47, 48] or decreased in lymph node metastases [46] and a high IGFBP-5/IGFBP-4mRNA expression ratio is related to decreased survival with poor prognosis [48, 49]. However, high IGFBP-5 expression has also been associated with increased OS [19] and reduced IGFBP-5 protein levels have been reported in the stroma surrounding aggressive metastatic BC [50]. In this respect it is interesting that a recent report demonstrated that co-culture of MCF-7 with carcinoma associated fibroblasts (CAFs) resulted in decreased IGFBP-5 mRNA expression in this BC cell line together with development of resistance to the SERD fulvestrant [51]. Further experiments with siRNA knock down of IGFBP-5 suggested a causal role for IGFBP-5 in the development of fulvestrant resistance. Whether deregulation of IGFBP expression is a causal factor in the development of other types of resistance (endocrine, chemotherapeutic) is a subject of ongoing investigation in our laboratories.

In conclusion, we have found that IGFBP-2 and -5 expression are reciprocally regulated following the acquisition of TamR in MCF-7 cells *in vitro*. The co- ordinate effects of these IGFBPs on tamoxifen resistance and cell migration respectively may provide a mechanism for the development of endocrine resistance and subsequent metastasis of BC epithelial cells.

## MATERIALS AND METHODS

### Tissue culture

A TamR derivative of MCF-7 was developed in house as previously described [52, 53]. Briefly, cells were cultured in phenol-red free RPMI 1640 supplemented with 5% dextran charcoal-stripped FCS (Invitrogen)–PR-DCS, 100 U/ml penicillin and 100 U/ml streptomycin and 100 nM 4-hydroxytamoxifen (4-HT; Sigma, Poole, UK) for 21-months during which 4-HT resistant variants developed. Isogenic controls were cultured in the same media, but with ethanol vehicle only. At 70–80% confluence cells were passaged (1:4) using 0.25% trypsin-EDTA (Invitrogen). Cell viability was determined by Trypan blue exclusion and cells were seeded at appropriate densities as described in the relevant figure legends. Cell line provenance was verified by annual STR profiling and bi-monthly mycoplasma testing (both in house).

### qRT-PCR

RNeasy^®^ Mini Kit (Qiagen, UK) was used to extract and purify mRNA exactly according to the manufacturer's protocol. High Capacity RNA to cDNA kit (Applied Biosystems, UK) was used according to the manufacturer's protocol to synthesise single stranded cDNA from 1 μg mRNA. qRT-PCR reactions were conducted in a final volume of 20 μl using TaqMan probes (Applied Biosystems). Expression of each gene was analysed in triplicate in 96 well plates. Non-template and reverse transcriptase negative controls were included. Taqman^®^ reactions were amplified using the Roche 480 LightCycler^®^. The amplification protocol included; 2 min at 50°C, 10 min at 95°C, and then 40 cycles of 2 step cycling; 15 sec at 95°C and 1 minute at 60°C. Quantification of PCR data was estimated based on the threshold cycle (C_t_). *RPLP0* was used as the housekeeping gene (HKGs) after validation. The data were analysed by using the comparative ΔC_T_ method (ΔCt (target) – ΔCt HKG) For calculating relative changes in gene expression (wt MCF7 v TamR) the ΔΔCt method was used where the fold change in gene expression is defined as 2^−ΔΔCt^ and is plotted as ordinate.

### Western and ligand blot

Conditioned media (CM: 1 ml) were freeze dried and stored at −20^°^C prior to analysis. Anti-hIGFBP-2 (MAB6741) and -5 (MAB8751) were from R & D Systems. MAB 6741 has < 1% cross reactivity with IGFBP-5; MAB 8751 has no cross reactivity with IGFBP-2. Anti-β-actin was from Santa Cruz Biotechnology. HRP conjugated rabbit anti-mouse was from Abcam (ab97046). Western blot protocols have been reported previously [54]. Ligand blotting with monobiotinylated IGF-2 (AMU010- GroPep) and streptavidin-HRP conjugate was as originally described [55]. Western and ligand blots were developed with Super-Signal^®^ West Femto Maximum Sensitivity Substrate (PN34095; Fisher Scientific) and images processed using Gel-Doc imager (Bio-Rad).

### Enzyme-linked immunosorbent assay (ELISA)

IGFBP-2 and IGFBP-5 concentrations in conditioned media were determined by ELISA using IGFBP-2 and IGFBP-5 DuoSet ELISA kits (Cat No DY674 and DY578 - R & D Systems) exactly according to the manufacturer's protocol. Assay range was 62.5–4000 pg/ml (IGFBP-2) and 125–8000 pg/ml (IGFBP-5). CM samples were diluted appropriately to fall within this range.

### IGFBP knockdown

A shRNA based strategy was used to knock down IGFBP-2 and IGFBP-5 expression in TamR and wt MCF-7 cells respectively. IGFBP-5 shRNA plasmid (sc-39591-sh), IGFBP-5 shRNA plasmid control (sc-108060), IGFBP-2 shRNA plasmid (sc-37195-sh), IGFBP-2 shRNA plasmid control (sc-108060) puromycin (CAY13884-25), shRNA plasmid transfection medium (sc-108062) and transfection reagent (sc-108061) (all Santa Cruz Biotechnology Inc., UK). Cells were transfected essentially according to the manufacturer's protocol. Briefly, cells (5 × 10^5^) were incubated in 6 well plates in PR-DCS at 37°C until 50–70% confluent. Cells were washed with 1 ml transfection media and incubated with 1 μg IGFBP or control shRNA plasmids and 0.5– 3% (v/v) transfection reagent in a final volume of 1ml for 6 hr. After a further 6 hr, 1 ml of PR- 10% DCS was added to each well and incubation continued overnight. Stable transfectants were selected in PR-DCS containing 6 μg/μl puromycin with media changes every 3–5 days. Puromycin resistant cells grew through at 3–4 weeks. Heterogeneous cell populations were expanded and after assay for IGFBP-2 and IGFBP-5 by ELISA cells were cloned by limiting dilution. After 2 weeks individual colonies were identifiable in a significant proportion of microtitre wells. Typically 6 such clones were allowed to grow to confluence and medium collected for IGFBP-2 or IGFBP-5 assay by ELISA. IGFBP concentrations in CM were compared to those obtained in contemporaneously grown wtMCF-7 and TamR cells and scrambled control shRNA clones. Clones which showed the highest level of knock down were subsequently expanded and stored as frozen stocks.

### Cell proliferation

Wt MCF-7, TamR, IGFBP-2 and IGFBP-5 knockdown or scrambled control shRNA clones were seeded in PR-DCS in 96 well plates at 5 × 10^3^ cells per well (100 μl suspension). After attachment overnight cells were washed with PBS and growth was monitored over the period 0–96 hr in PR-DCS ± 1 uM 4-hydroxy tamoxifen (4 HT).

### Incucyte cell migration assay

Cells were seeded in 96-well Essen Image Lock plates at 5 × 10^5^ per well in PR-DCS. Plates were incubated for 18 h to allow cell attachment following which sterilised 96-well Wound-Maker pins were used to simultaneously generate precise and reproducible cell-free zones 700–800 μm wide in cell monolayers. Plates were placed in the IncuCyte incubator at 37°C and equilibrated for a minimum of 15 minutes before the first scan. IncuCyte software acquires images in real time at 1 hr intervals for the duration of the experiment (96 h) and integrated software quantifies cell migration using the metric relative wound density (RWD). Migration of six different cell lines was analysed using this technique – parental wt and TamR MCF-7 cells; IGFBP-2 KO (clone F8) and scrambled shRNA control; IGFBP-5 KO (clone B4) and scrambled shRNA control. Data are presented along as plots of RWD v time. For each cell line 12 replicates were performed and data are generated as mean ± SD.

### Immunohistochemistry

#### Tissue microarray (TMA) construction and immunohistochemistry

Ethical approval was obtained from the Leeds (East) Local Research Ethics Committee at St James's University Hospital, Leeds, UK (06/Q1206/180). TMAs were constructed previously from an initial series of 351 cases of primary operable invasive breast carcinoma from patients presenting from 1987–2005 all of whom had received TAM and comprised, initially, 108 and 243 cases who did or did not experience a relapse, respectively [56]. As this series has been used extensively in other publications [57–60] some of the TMA cores were exhausted, meaning that in this study 77 relapsed and were designated clinically TamR (cTamR) while 193 did not relapse and were designated clinically TamS (cTamS) were available for study. Mean follow up time for the former was 87 months (range 11–199) and the latter 101 months (range 7–214). Survival in the cTamR cohort was updated in June 2015 and the cTamS cohort in July 2013. The TamS cohort were updated 2 years earlier than the resistant patients and some may have relapsed in the subsequent 2 years however as indicated above the median follow up was actually longer for the TamS cohort than the TamR cohort (101 and 87 months, respectively). Patients were censored at the last day they were known to be alive. Histopathological details are presented in Supplementary Table S1A and S1B. The cTAMs patients had lower grade, smaller tumours and a higher proportion of IGFBP-2 positive tumours (Supplementary Table S1A). The IGFBP-2 positive tumours were smaller than the IGFBP-2 negative tumours (Supplementary Table S1B).

Using the Human Protein Atlas (http://www.proteinatlas.org/), suitable IGFBP-2 and IGFBP-5 antibodies were identified (Abcam-ab109284; and Santa Cruz SC-6006) respectively. These antibodies do not cross react with the other IGFBPs. Negative controls (primary antibody omitted) were also included. Antigen retrieval was achieved after de-waxing using Mena Path Revelation buffer solution (cat no MP-607-X500) in the Mena Path pressure cooker containing 500 ml distilled water for 40 minutes. Immediately following this slides were immersed in PBST (1× TBS containing 0.2% Tween 20) buffer then distilled water. Then, slides were washed in PBST and blocked with 100 μl Novacastra peroxidase (cat no RE7101) for 10 minutes, washed with PBST, then 100 μl of protein blocking solution (cat no RE7102) was added and incubated for 2 minutes (both incubations at room temperature). Rabbit monoclonal antibodies against hIGFBP-2 (1:50 v/v) and goat polyclonal antibody against IGFBP-5 (1:50 v/v) were added and incubated at room temperature for 1 hour. Slides were washed 3 times for 5 minutes with PBST and 100 μl of secondary antibody (Novolink polymer cat no RE7112) was added and incubated for 30 minutes. After washing 3 times in PBST for 5 minutes 100 μl of the diluted DAB chromogen (1:20 v/v;) cat no RE7105) was added to each slide and incubated at room temperature RT (5 minutes). Rehydration was achieved through graded alcohols, 100%, 75%, 50%, and 25% f (3 minutes in each), and washed in running tap water. Endogenous peroxidase activity was blocked (0.75% H_2_O_2_, 20 minutes) and then rinsed in running tap water. Slides were counter stained by immersing in Mayer's haematoxylin for 1 minute, washed in running tap water, then immersed in Scott's tap water for 2 minutes followed by a further wash in running tap water. The slides were then dehydrated in a series of ethanols (25% for 15 seconds, 50% for 2 minutes, 70% for 5 minutes, and 100% for 5 minutes) then immersed in xylene 3 times for 3 minutes. [65] Finally, slides were mounted in DPX and cover-slips applied. Subsequently, slides were scanned (Aperio ScanScope^®^) and scored using a pixel intensity algorithm (Aperio) A Receiver Operating Characteristic Curve (ROC) was used to determine the optimal cut-off point using an online program (http://molpath.charite.de/cutoff/; [61].

#### *In silico* analysis

An online survival analysis tool was used to study the relationship between IGFBP-5 and IGFBP-2 expression and overall survival (Kaplan Meir Plotter; http://kmplot.com/analysis/ (accessed 9 Nov 2015). This data set comprises 3557 patients with overall survival data on 1117 patients As the focus had been on tamoxifen sensitivity and resistance, we selected a cohort of 65 patients with IGFBP-5 and IGFBP-2 expression data (classified as either above or below the median) who had received tamoxifen therapy, irrespective of ER status and whether they received chemotherapy.

### Statistics

Data were analysed for significant differences using Student's unpaired *t*-test (ELISA) or repeated measures ANOVA followed by Bonferroni's post-hoc test. Fisher's exact test was performed to demonstrate relationships between immunohistochemical findings and clinicopathological variables. Survival durations in months were calculated using Statistical Package for Social Sciences (SPSS) version 22 software and Log-rank test (Mantel–Cox test) test was used to plot Kaplan-Meier survival curves.

## SUPPLEMENTARY MATERIALS FIGURES AND TABLES


