# Supplementary material for: Deregulation of IGF-binding proteins -2 and -5 contributes to the development of endocrine resistant breast cancer in vitro
Source: Oncotarget. 2016 Apr 1;7(22):32129–43. doi: 10.18632/oncotarget.8534 (PMC5078002; doi:10.18632/oncotarget.8534)
Supplement: Supplementary file 1 [file oncotarget-07-32129-s001.pdf]

Deregulation of IGF-binding proteins -2 and -5 contributes to the development of endocrine resistant breast cancer *in vitro*

Supplementary Materials

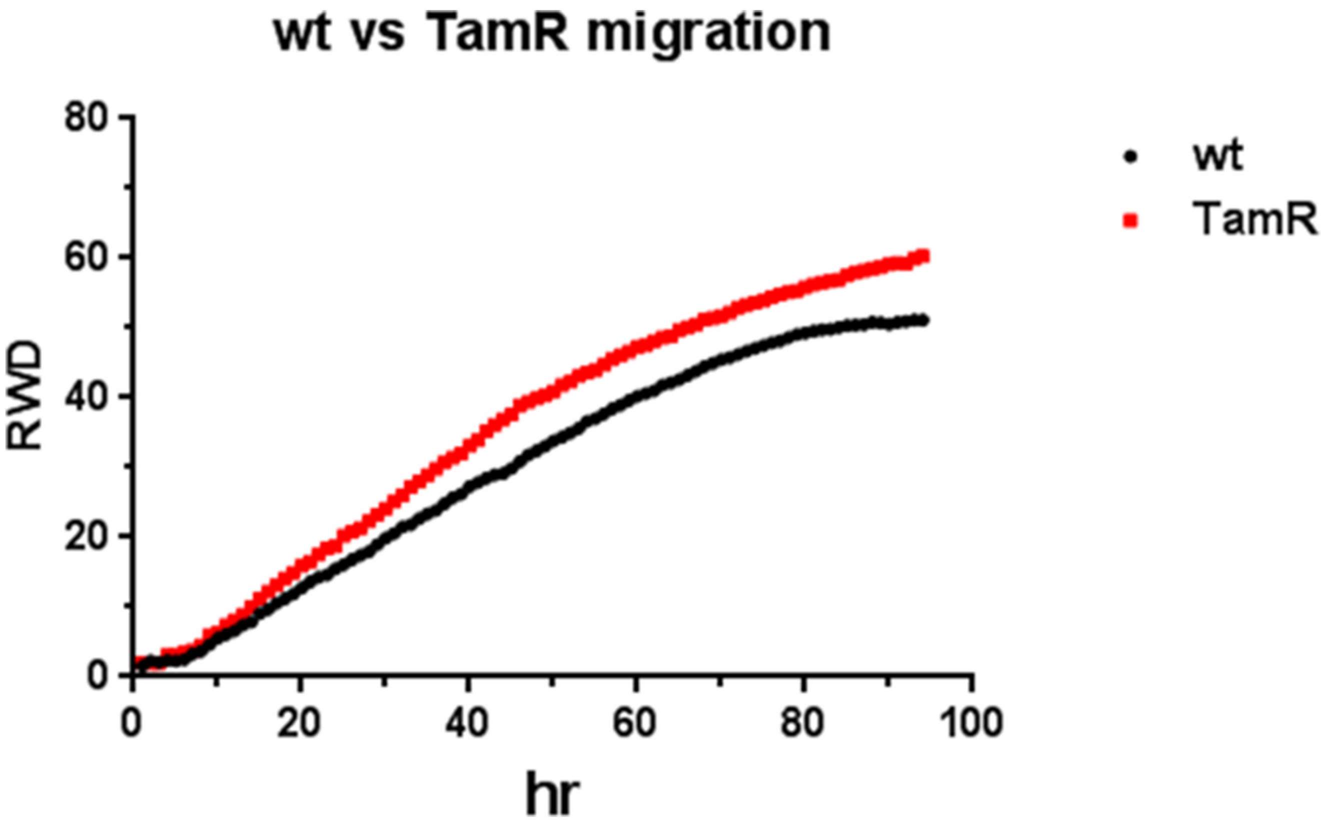

Supplementary Figure S1: A real time analysis of relative wound density (RWD) for MCF-7 and TamR. Cell migration was monitored over the period 0–94 hr. The black curve represents wt and the grey curve represents TamR MCF-7 cells. Data are presented as mean ± SD; $n = 12$ for each time point. This experiment was performed twice with similar results in each instance and a representative experiment is shown. Curves were analysed by repeated measures ANOVA followed by Bonferroni's post-hoc test. $p < 0.0001$ wt v TamR GraphPad Prism 5.0.

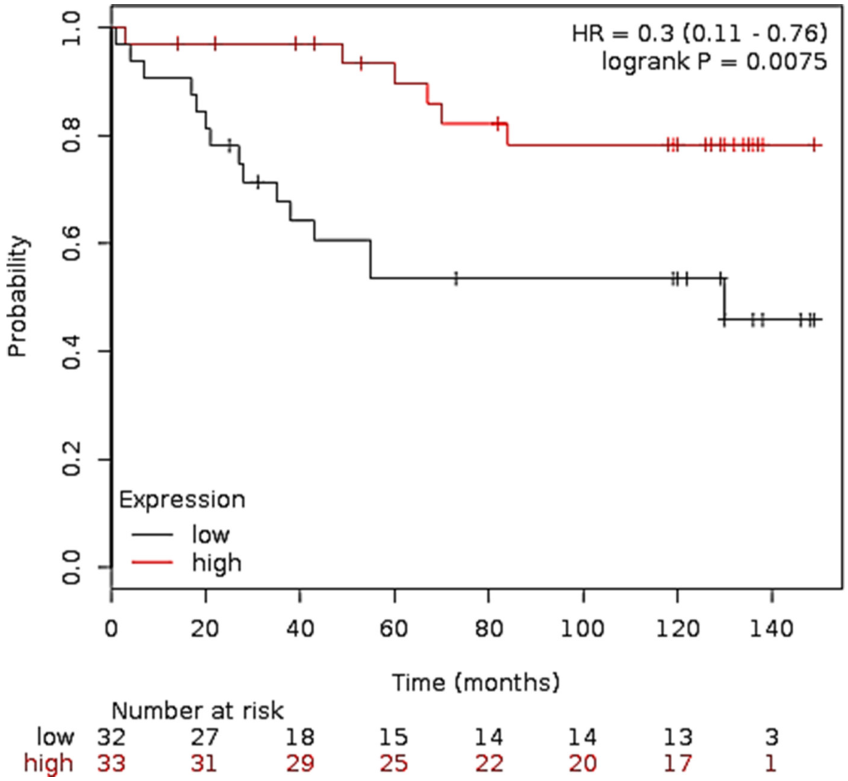

Supplementary Figure S2: IGFBP-5 gene expression data mined from a publically available resource (http://www.kmplot.com; [62]. KMPlot indicated expression of IGFBP-5 was associated with improved overall survival.

Supplementary Table S1A: Clinico-pathological parameters for Tamoxifen sensitive vs Tamoxifen resistant

	cTamS *n* = 193 (%)	cTamR *n* = 77 (%)	*P*
ERα			
–	24 (12.5)	13 (16.9)	0.23
+	167 (86.5)	55 (71.4)	
Unknown	2 (1)	9 (11.7)	
Grade			
1	49 (25.4)	8 (10.4)	< 0.001
2	107 (55.4)	28 (36.4)	
3	37 (19.2)	35 (45.5)	
Unknown	0	6 (7.8)	
Size			
T1	82 (42.5)	17 (22.1)	0.012
T2	50 (25.9)	25 (32.5)	
T3	9 (4.7)	7 (9.1)	
Unknown	52 (26.9)	28 (36.3)	
LN			
–	82 (42.5)	21 (27.3)	0.18
+	109 (56.5)	43 (55.8)	
Unknown	2 (1)	13 (16.9)	
IGFBP2			
–	59 (30.6)	36 (46.8)	0.016
+	134 (69.4)	41 (53.2)	

Supplementary Table S1B: Relationship between IGFBP-2 expression and clinico-pathological parameters

Parameter	cTamS (*n* = 193) IGFBP-2 (%)		P	cTamR (*n* = 77) IGFBP-2		*P*	Combined cohort (*n* = 270) IGFBP-2		*P*
	–	+		–	+		–	+	
ERα									
–	9 (15.3)	15 (11.2)	0.48	6 (16.7)	7 (17.1)	1.0	15 (15.8)	22 (12.6)	0.46
+	49 (83)	118 (88.1)		27 (75)	28 (68.3)		76 (80)	146 (83.4)	
Unknown	1 (1.7)	1 (0.7)		3 (8.3)	6 (14.6)		4 (4.2)	7 (4)	
Grade									
1	15 (25.4)	34 (25.4)	0.63	5 (13.9)	3 (7.3)	0.68	20 (21.1)	37 (21.1)	0.99
2	35 (59.3)	72 (53.7)		13 (36.1)	15 (36.6)		48 (50.5)	87 (49.7)	
3	9 (15.3)	28 (20.9)		16 (44.4)	19 (46.3)		25 (26.3)	47 (26.9)	
Unknown	0	0		2 (5.6)	4 (9.8)		2 (2.1)	4 (2.3)	
Size									
T1	22 (37.3)	60 (44.8)	0.22	4 (11.1)	13 (31.7)	0.007	26 (27.4)	73 (41.7)	0.038
T2	20 (33.9)	30 (22.4)		18 (50)	7 (17.1)		38 (40)	37 (21.2)	
T3	4 (6.8)	5 (3.7)		3 (8.3)	4 (9.7)		7 (7.4)	9 (5.1)	
Unknown*	13 (22)	39 (29.1)		11 (30.6)	17 (41.5)		24 (25.3)	56 (32)	
LN									
–	20 (33.9)	62 (46.3)	0.15	9 (25)	12 (29.3)	0.59	29 (30.5)	74 (42.3)	0.61
+	38 (64.4)	71 (53)		23 (63.9)	20 (48.8)		61 (64.2)	91 (52)	
Unknown	1 (1.7)	1 (0.7)		4 (11.1)	9 (21.9)		5 (5.3)	10 (5.7)	

For size unknown cTamS cohort there was 2 multifocal tumours in the BP2+ group and one in the BP2– group.
